# Efficient electroreduction of CO_2_ to C_1_ and C_2_ products using atomically dispersed boron N–C@graphite catalysts

**DOI:** 10.1039/d5ya00260e

**Published:** 2025-10-22

**Authors:** Farzaneh Yari, Simon Offenthaler, Sankit Vala, Dominik Krisch, Wolfgang Schöfberger

**Affiliations:** a Institute of Organic Chemistry, Laboratory for Sustainable Chemistry and Catalysis (LSusCat), Johannes Kepler University Linz Altenberger Straße 69 4040 Linz Austria wolfgang.schoefberger@jku.at https://www.jku.at/en/institute-of-organic-chemistry/team/schoefberger-lab

## Abstract

Atomically precise control of active sites is essential for advancing metal-free electrocatalysts for the CO_2_ reduction reaction (CO_2_RR). We report boron- and nitrogen-co-doped graphite (boron–N–C@graphite) derived from chloro-boron subphthalocyanine (Cl-B-SubPc), an aromatic macrocyclic precursor that directs simultaneous incorporation of B and N into conductive carbon frameworks. X-Ray photoelectron spectroscopy reveals the formation of B–C and B–N motifs alongside pyridinic and graphitic N, generating electron-deficient centers that modulate intermediate binding energies. The resulting catalysts display pronounced structure–activity correlations: pyrolysis at 800 °C favors formate and acetate formation, whereas 1000 °C yields a more graphitic catalyst with enhanced CO selectivity (faradaic efficiency up to 26.9%). Mechanistic analysis indicates that the B–N synergy stabilizes *CO_2_-intermediates, suppresses hydrogen evolution, and enables C–C coupling. Both catalysts exhibit long-term stability (>180 h), and in zero-gap electrolyzers deliver industrially relevant current densities (150 mA cm^−2^) with CO faradaic efficiencies of 79.0% and 87.4%, respectively. These findings establish B,N-*co*-doped carbons from molecular precursors as a versatile platform for elucidating active-site chemistry and for guiding the rational design of sustainable, high-performance CO_2_RR catalysts.

## Abstract 2

Boron NC@graphite catalysts function as efficient, noble-metal-free electrocatalysts for CO_2_ conversion to CO and C_1_/C_2_ products. Boron subphthalocyanines represent a new, scalable class of molecular electrocatalysts.

## Graphical abstract

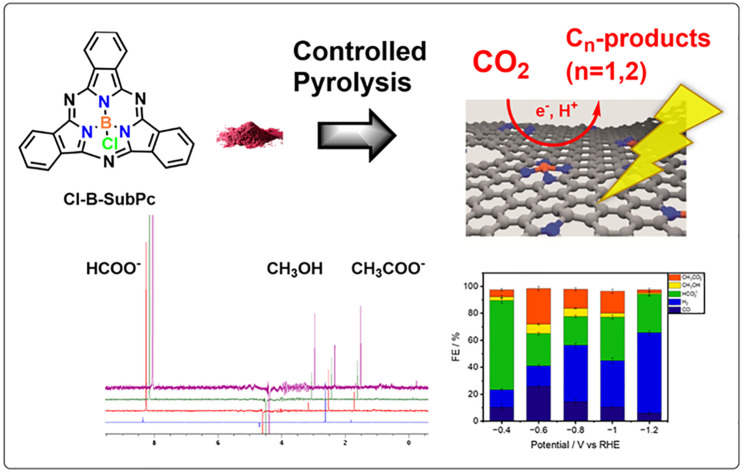

## Introduction

Central to the success of CO_2_RR is the design of efficient electrocatalysts that exhibit high activity, selectivity, and durability under operating conditions. Traditionally, noble metal catalysts such as Au, Ag, and Pd have shown excellent performance, but their high cost and scarcity limit widespread application.^1–6^

This has driven the search for noble metal-free multifunctional electrocatalysts that offer both high selectivity and high current density for CO production. In this regard, carbon-based metal-free catalysts (CMFCs) have attracted significant attention due to their natural abundance, low cost, excellent chemical stability, and environmental benignity.^7,8^ A diverse range of CMFCs have been explored for CO_2_RR, including heteroatom-doped carbons where elements such as nitrogen (N), boron (B), fluorine (F), and sulfur (S) are introduced into the carbon lattice and defect-rich carbons with engineered vacancies or edges.^9–11^ Such doping introduces active sites and modifies the electronic structure of the carbon framework, enhancing CO_2_ adsorption and catalytic reduction.

Among heteroatom-doped carbons, materials such as N-doped graphene, N-doped carbon nanotubes, N-doped carbon nanofibers, B-doped nanodiamonds, B,N co-doped nanodiamonds, and N,S co-doped porous carbon nanofiber membranes have demonstrated remarkable electrocatalytic performance for the conversion of CO_2_ to products including CO, formic acid (HCOOH), methane (CH_4_), and ethanol (C_2_H_5_OH).^12–15^ Particularly, B,N co-doped carbons have shown enhanced catalytic activity attributed to a synergistic effect between boron and nitrogen dopants, which tunes the electronic properties and optimizes intermediate adsorption energies during CO_2_RR. However, the detailed mechanistic understanding of this synergy remains incomplete. Moreover, the influence of porous structure combined with B,N co-doping on the overall catalytic performance has been scarcely studied. Therefore, developing B,N co-doped porous carbon materials and investigating their structure–property relationships for CO_2_RR is highly desirable.

Chloroboron subphthalocyanine (Cl-B-SubPc), a boron-containing aromatic macrocycle,^16^ presents an ideal molecular precursor for synthesizing B,N co-doped carbon electrocatalysts. Its unique bowl-shaped structure, with a central boron atom coordinated to nitrogen-rich aromatic rings and a chlorine ligand, provides a rich source of boron and nitrogen atoms for incorporation into a carbon matrix upon pyrolysis.^12^ However, as a molecular catalyst, Cl-B-SubPc suffers from limited stability and challenges in electrode integration, restricting its practical use in CO_2_RR applications. To overcome these limitations, pyrolysis of Cl-B-SubPc under inert atmosphere is employed to convert the molecular precursor into a porous carbon material co-doped with boron and nitrogen. Pyrolysis induces carbonization of the organic framework, partial retention of nitrogen functionalities, and embedding of boron atoms into the carbon lattice, resulting in a conductive, porous material with abundant active sites for CO_2_ reduction. The process also generates hierarchical porosity, enhancing electrolyte access and mass transport to catalytic centers.

During pyrolysis, nitrogen atoms are transformed into various species such as pyridinic, pyrrolic, and graphitic nitrogen, each contributing differently to catalytic activity by altering local electronic structures and facilitating proton–electron transfer steps. Boron atoms integrate as B–N–C motifs or B–C bonds, inducing electron-deficient centers that stabilize CO_2_ adsorption and reaction intermediates. The synergy between B and N dopants in this carbon network creates optimized active sites that lower the activation energy for CO_2_ reduction, enhancing catalytic efficiency and selectivity towards CO production.

Recent studies highlight that CMFCs derived from molecular precursors, especially those co-doped with boron and nitrogen, achieve high current densities and selectivities comparable to noble metals, along with excellent long-term stability. This renders them promising candidates for scalable, cost-effective CO_2_RR catalysts. Building on this, we investigate the electrocatalytic performance of a boron nitrogen doped graphite material (boron–N–C@graphite) synthesized *via* pyrolysis of Cl-B-SubPc. Comprehensive characterization techniques including X-ray photoelectron spectroscopy (XPS), is employed to elucidate the bonding environment and microstructure. Electrochemical tests are conducted to evaluate CO_2_RR activity, product selectivity, and stability.

This work aims to deepen the mechanistic understanding of B,N co-doped carbon catalysts and establish design principles for molecular precursor-derived metal-free electrocatalysts. Ultimately, such catalysts could enable sustainable, efficient conversion of CO_2_ to valuable chemicals, contributing to carbon-neutral energy cycles and environmental remediation.

## Experimental section

### Materials and methods

All chemicals were purchased from Alfa Aesar, Sigma-Aldrich, Strem, BLDpharm, TCI, or Merck, and used without further purification unless otherwise stated. Anhydrous tetrahydrofuran (THF) and dichloromethane (DCM) were dried and dispensed under an inert atmosphere using a solvent purification system (MB-SPS-7, M. Braun Inertgas-Systeme GmbH) equipped with activated molecular sieves and operated under argon.

X-ray photoelectron spectroscopy (XPS) measurements of the synthesized complexes were conducted using a Theta Probe system (Thermo Fisher Scientific) equipped with a monochromated Al Kα X-ray source (1486.6 eV). Instrument control, data acquisition, and spectral analysis were performed using the Avantage software suite provided by the manufacturer. XPS characterization of electrode samples was carried out using a Nexsa G2 XPS system (Thermo Fisher Scientific) at the ZONA facility. This instrument allows for high-resolution surface analysis, providing elemental composition and chemical state information within the top 5–10 nm of the material surface, depending on the sample matrix.

## Results and discussion

### Synthesis of molecular catalyst

Chloro-boron subphthalocyanine (Cl-B-SubPc) precursor (0.50 g, 1.6 mmol) was dissolved in mesitylene (20–30 mL) under an argon atmosphere.^17^ Boron trichloride (7.5 mL, 1.0 M in CH_2_Cl_2_) was added dropwise, and the mixture was refluxed for 2 h. Reaction progress was monitored by TLC (*n*-heptane/CH_2_Cl_2_, 1 : 3). Upon completion, the solvent was removed under reduced pressure, and the crude product was dried in a desiccator. The solid was washed sequentially with toluene and methanol, then purified *via* silica gel column chromatography (heptane:ethyl acetate, 3 : 1, *R*_f_ = 0.68) to afford the desired Cl-B-SubPc as a purple solid. Yield: 73%.

### Synthesis of catalysts

**Fig. 1:**
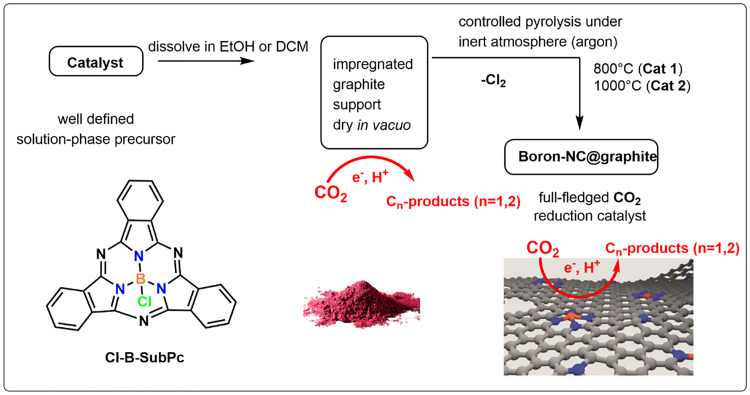
Boron subphthalocyanine molecular precursor and the two-step synthesis of the boron–NC@graphite.

A one-step pyrolysis process was employed to synthesize activated boron N–C@graphite catalysts. Following collection, the catalyst precursor powder was carefully transferred into a quartz boat, which was then centrally positioned within a quartz tube reactor. To eliminate any residual air or moisture, the system was thoroughly purged with high-purity argon gas for 30 minutes under a constant flow. After purging, the sample underwent a rapid thermal treatment, wherein the temperature was swiftly elevated to either 800 °C (Cat 1) or 1000 °C (Cat 2) using a previously reported heat-shock procedure. This high-temperature pyrolysis step was sustained for one hour under continuous argon flow to ensure proper transformation of the precursor material. Upon completion of the heat treatment, pyrolysis was promptly terminated by removing the quartz tube from the furnace, allowing the system to cool naturally to ambient temperature while maintaining an inert argon atmosphere to prevent oxidation. Once the tube and contents had fully cooled, the catalyst sample was collected for subsequent characterization and analysis (Fig. 1).^23^

### Catalyst characterization

The catalyst loading was calculated by measuring the electrode mass before and after spray-coating, resulting in a loading of approximately 0.2 mg cm^−2^. For X-ray photoelectron spectroscopy (XPS) analysis, the gas diffusion layers (GDLs) were prepared using the same procedure, except without the addition of Nafion. This modification was made to prevent potential interference from Nafion's carbon and oxygen content, which could obscure the detection of other elemental signals in the XPS spectra.

**Fig. 2:**
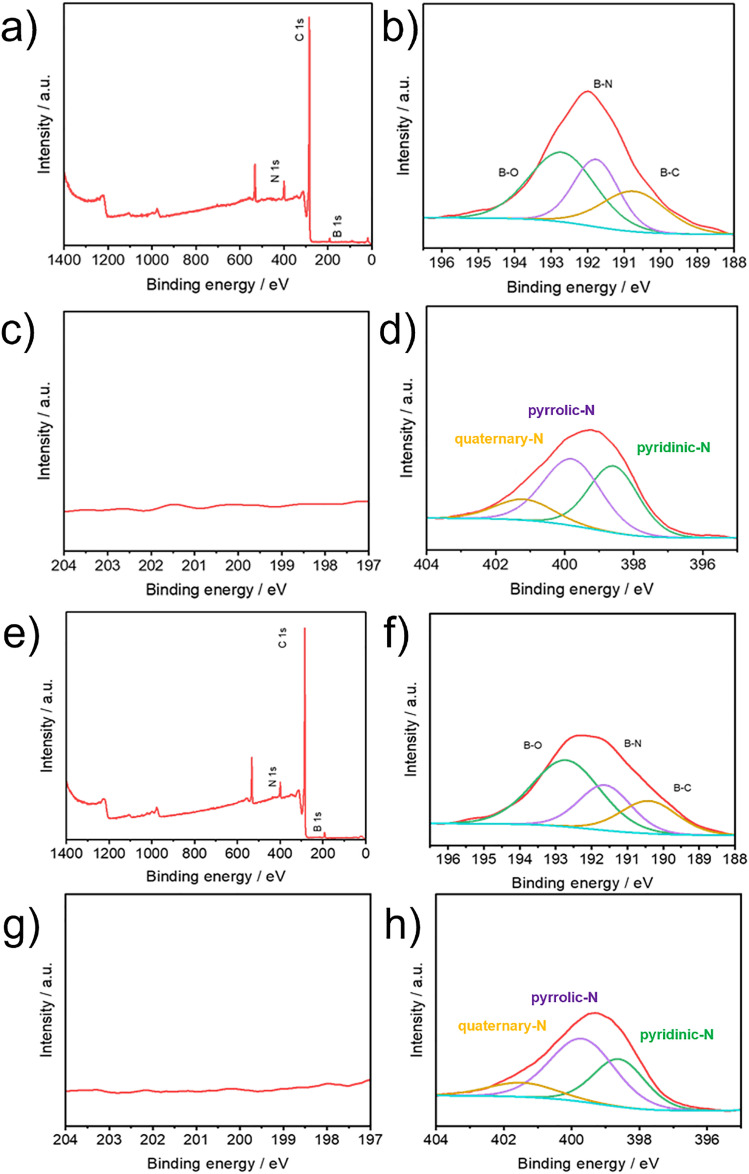
X-ray photoelectron spectra of the (Cl-B-SubPc)/CB (800 °C, Cat 1) (a) full-energy scan, (b) B1s, (c) Cl2p, (d) N1s and of the (Cl-B-SubPc)/CB (1000 °C, Cat 2) (e) full-energy scan, (f) B1s, (g) Cl2p and (h) N1s.

XPS was performed to verify the successful pyrolysis of the Cl-B-SubPc. Elemental analysis revealed that both samples contain B, C, N, and O, while Cl was detected exclusively in the non-pyrolyzed material, where the B/Cl ratio = 1 (Fig. S1). The absence of Cl in the pyrolyzed sample indicates its removal during thermal treatment. The narrow scan XPS spectra of B 1s and Cl 2p for Cl-B-SubPc at room temperature display a B 1s binding energy peak at 192.0 eV, indicating the presence of boron species (Fig. 2). The N 1s peak at 399.5 eV corresponds to the =N– species in Cl-B-SubPc (Fig. 2(b) and (f)), while the peak at 398.7 eV is attributed to nitrogen coordinated to the central B–N structure.

In the B 1s region of Cat 1 and Cat 2, the spectrum shows three resolved peaks at binding energies of 189.32, 189.85, and 191.15 eV. These are attributed to B–C, B–N, and B–O interactions, suggesting a chemically diverse boron environment within the structure. The high-resolution N 1s spectrum of Cat 1 and Cat 2 (Fig. 2(d) and (h)) can be deconvoluted into three main nitrogen species with shifting around 2.0 eV toward higher binding energy. A peak at 397.8 eV is attributed to N–B bonding, characteristic of the boron coordination environment. The component at 400.0 eV corresponds to N–C bonding, which includes contributions from pyridinic and pyrrolic nitrogen. Finally, a peak observed at 401.4 eV is assigned to graphitic nitrogen, suggesting the presence of more ordered carbon–nitrogen domains. This shift is characteristic of nitrogen species embedded in more graphitic environments, indicating increased structural ordering and electronic delocalization due to pyrolysis. The relative decrease in B–N bonding in Cat 2 (1000 °C) compared to Cat 1 (800 °C) arises from the higher pyrolysis temperature, which promotes graphitization and favors the formation of B–C bonds over B–N (Fig. 2(d) and (h)). The XRD patterns of the obtained samples at 800 and 1000 °C are shown in Fig. S3. All samples show two weak diffraction peaks centered at 2*θ* = 23.2° and 43°, respectively. The value of *d*_002_ is about 0.36 nm, larger than that of graphite (0.335 nm), implying a random combination of graphitic and turbostratic stacking. In addition, with increasing calcination temperature, the intensities of the 2*θ* peaks at approximately 43° increases, due to the formation of higher degree of graphitic structure at higher carbonization temperature, which will greatly improve the electrical conductivity.

Fig. S2 illustrates the XPS results obtained after electrocatalysis, revealing no major changes in the chemical composition and coordination environment of the catalysts evolve during operational conditions.

### Electrode preparation

To prepare the catalyst ink, 2 mg of the synthesized catalyst powder was accurately weighed and dispersed in 2 mL of methanol under ambient conditions. To this suspension, 20 μL of a 5 wt% Nafion® 117 solution was introduced as a binder, which aids in enhancing the interfacial adhesion between the catalyst and the substrate, while also promoting better film formation. The mixture was then subjected to ultrasonication in a bath sonicator for 30 minutes. This step was essential to achieve a homogeneous dispersion of the catalyst particles throughout the solvent matrix, ensuring consistent ink quality for electrode fabrication.

Following ink preparation, the well-dispersed suspension was uniformly spray-coated onto a defined 1 × 1 cm area of carbon paper (TGP-H-60, Thermo Scientific), which served as the substrate for electrochemical characterization conducted in a conventional H-type cell (Fig. S4). For experiments involving a zero-gap electrolyzer setup, gas diffusion electrodes (CeTech, W1S1011, thickness: 365 μm) were employed as the support. After coating, all prepared electrodes were dried under vacuum conditions overnight to facilitate complete evaporation of the solvent and to preserve the mechanical stability and integrity of the catalyst layer prior to electrochemical measurements.

### Electrochemical measurements

**Fig. 3:**
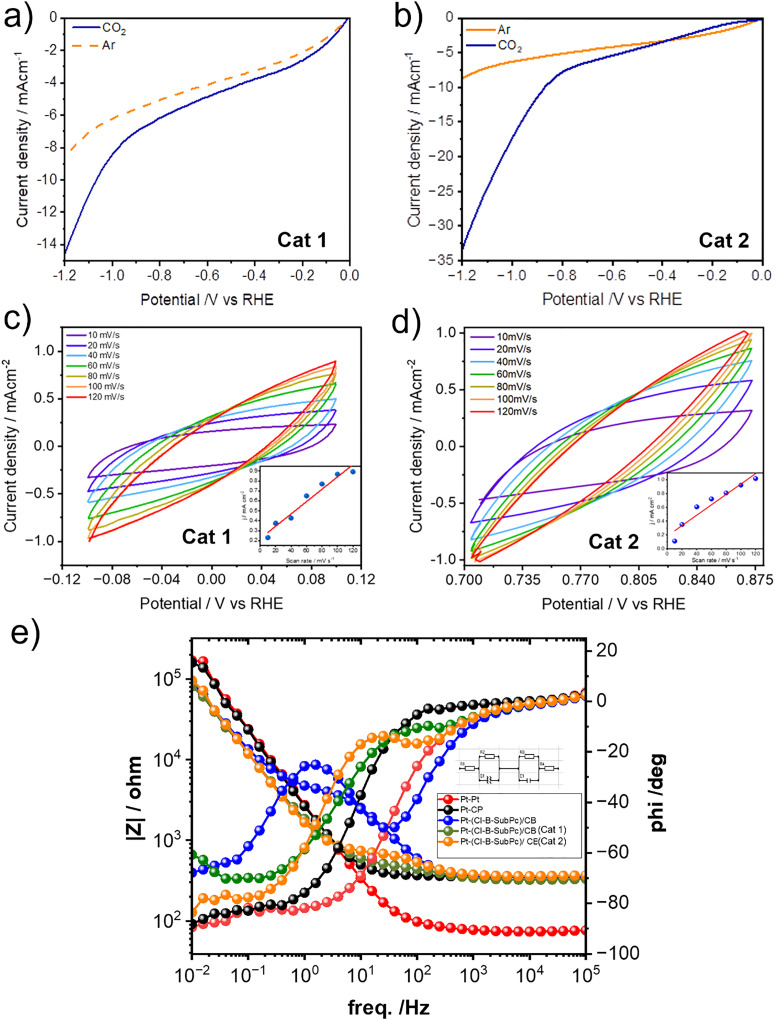
(a) LSV curves at a scan rate of 30 mV s^−1^ (Cl-B-SubPc)/CB (800 °C, Cat 1)/CB WEs with Ar and CO_2_ saturation 0.1 M CsHCO_3_. (b) LSV curves at a scan rate of 30 mV s^−1^ (Cl-B SubPc)/CB (1000 °C, Cat 2) WEs with Ar and CO_2_ saturation 0.1 M CsHCO_3_. (b) Cyclic voltammograms of (Cl-B-SubPc)/CB (800 °C, Cat 1) at different sweep rates of 10, 20, 40, 60, 80, 100, and 120 mV s^−1^ in 0.1 M CsHCO_3_ and a linear plot of capacitive current *versus* scan rate. (c) Cyclic voltammograms of (Cl-B-SubPc)/CB (1000 °C, Cat 2) at different sweep rates of 10, 20, 40, 60, 80, 100, and 120 mV s^−1^ in 0.1 M CsHCO_3_ and a linear plot of capacitive current *versus* scan rate. (e) Bode plot recorded *via* electrochemical impedance spectroscopy of (Cl-B-SubPc)/CB, (Cl-B-SubPc)/CB (800 °C, Cat 1), and (Cl-B-SubPc)/CB (1000 °C, Cat 2) catalyst modified carbon paper electrode in the frequency range of 1 × 10^−1^ Hz to 1 × 10^5^ Hz with a perturbation amplitude of 10 mV.

The electrochemical performance of the synthesized electrocatalysts was thoroughly evaluated using heterogeneous techniques. Due to the solid, pyrolyzed nature of the catalysts, homogeneous methods were not applicable, as the materials are insoluble and cannot be dispersed at the molecular level in solution-phase systems. Therefore, all characterizations were conducted in configurations where the catalyst remained immobilized on an electrode surface, ensuring relevance to practical, device-level applications. To accurately capture electrochemical behavior under conditions relevant to real-world applications, all measurements were conducted using heterogeneous systems, where the catalyst remained immobilized on the electrode surface. This approach enables the assessment of practical performance metrics and interfacial phenomena that are representative of scaled-up electrocatalytic processes. Linear sweep voltammetry (LSV) was employed as the primary technique to evaluate catalytic activity (Fig. 3(a), (b) and Fig. S5, S6). The measurements were performed in an aqueous 0.1 M cesium bicarbonate (CsHCO_3_) electrolyte, chosen specifically for its ability to suppress hydrogen evolution, thereby minimizing competing side reactions. Prior to CO_2_ saturation, the CsHCO_3_ solution was purged with ultra-high-purity argon gas (99.99%) for 15 minutes to remove dissolved oxygen and other gaseous impurities that could interfere with the electrochemical response.

Following deoxygenation, the electrolyte was saturated with high-purity CO_2_ gas (99.99%) at a controlled flow rate of 10 mL min^−1^ for approximately one hour. The saturation process was monitored until the solution pH stabilized at 6.8, consistent with literature values for CO_2_-saturated CsHCO_3_ solutions under ambient conditions.^18^ This careful electrolyte preparation ensured a reproducible and well-defined reaction environment for all subsequent electrochemical measurements.

The experiments were performed at room temperature. Cat 1 and Cat 2 both demonstrated significantly higher current densities in a CO_2_-saturated electrolyte compared to an Ar-saturated environment, clearly indicating their electrocatalytic activity toward CO_2_ reduction. This enhanced current response under CO_2_ suggests that both catalysts actively participate in the electrochemical conversion of CO_2_, likely facilitating key reduction steps that are absent in the inert Ar atmosphere. These observations highlight the potential of Cat 1 and Cat 2 as promising candidates for CO_2_ electroreduction applications.

In the aqueous electrolyte system, the total current observed during CO_2_ electroreduction (e-CO_2_R) both involves contributions of the CO_2_ reduction reaction (CO_2_RR) and the competing hydrogen evolution reaction (HER), and hence it makes difficult to unambiguously assign specific features in the linear sweep voltammetry (LSV) profiles to either processes.

### Electrochemically active surface area (ECSA)

The electrochemically active surface area (ECSA) provides an important quantitative measure of the accessible surface area available for electrochemical reactions. In this study, ECSA was estimated *via* the measurement of the double-layer capacitance (*C*_DL_), which reflects the capacitive charging of the electrode/electrolyte interface in a non-faradaic region, where no redox reactions occur. To determine *C*_DL_, cyclic voltammetry (CV) was conducted in a narrow potential window where only double-layer charging occurs. For Cat 1, the CV was recorded over a 200 mV window centered at −0.02 V *vs.* RHE, while for Cat 2 (Fig. 3(c), (d) and Fig. S7), the window was centered at 0.10 V *vs.* RHE. All potentials were referenced to the reversible hydrogen electrode (RHE) using the standard conversion:*E* (V *vs.* RHE) = *E* (V *vs.* Ag/AgCl) + 0.197 + 0.059 × pHCV scans were performed at varying scan rates of 5, 10, 25, and 50 mV s^−1^ to capture the relationship between capacitive current and scan rate.

The different current densities (*i*_c_, mA cm^−2^) were plotted as a function of scan rate (*v*, mV s^−1^) with a slope equal to the *C*_DL_ (μF cm^−2^).ECSA (cm^2^) = *C*_DL_/*C*_ref_*C*_DL_ = *C*_DL_ × *S* (*S* is surface area of electrode, cm^−2^)

The ECSA can be obtained by comparing the correlation *C*_DL_ (μF) to a smooth planar surface (*C*_REF_, μF cm^−2^) which was assumed to be 68.2 μF cm^−2^ following these equations. The reference capacitance (*C*_REF_ = 68.2 μF cm^−2^) was chosen following established values for carbon-based electrodes.^19^ We emphasize that the calculated ECSA values are relative comparisons rather than absolute values. This approach remains valid for comparing Cat 1 and Cat 2 under identical conditions, while acknowledging that absolute values may differ with more material-specific standards. Based on this approach, the ECSA values for Cat 1 and Cat 2 were determined to be 90.9 and 110.4 cm^2^, respectively.

### Electrochemical impedance spectroscopy (EIS)

Electrochemical impedance spectroscopy (EIS) was employed to gain insights into the charge transport and interfacial properties of the electrochemical system during CO_2_ reduction. Impedance spectra were recorded over a frequency range from 10^6^ Hz to 0.01 Hz with an AC amplitude of 10 mV (Fig. 3(e)). This technique was specifically used to investigate the catalytic behavior of two electrode materials, designated as Cat 1 and Cat 2.

To establish a baseline for the system's resistance, control experiments were first conducted using a symmetric cell configuration comprising two platinum electrodes immersed in 0.1 M CsHCO_3_. This setup enabled the determination of the inherent resistance of the electrolyte. The system was then transitioned into a two-compartment H-type electrochemical cell separated by a Nafion membrane to isolate and subtract the membrane's resistance from the total impedance response, allowing for a more accurate evaluation of electrode performance.

In a subsequent series of measurements, one of the platinum electrodes was replaced with unmodified carbon paper, which was then further modified with Cat 1 and Cat 2 to serve as the working electrode in separate experiments. These modified electrodes were evaluated under identical conditions to assess the full electrochemical response and quantify resistive and capacitive elements within the system.

The resulting Bode plots, shown in Fig. 3(e) and Fig. S8, provide a comprehensive overview of the frequency-dependent behavior of the cell. Corresponding fitted impedance values, including solution resistance (*R*_s_), charge transfer resistance (*R*_ct_), and other circuit elements, are compiled in Table 1. A detailed analysis of the resistive contributions from individual components namely, the working electrode, Nafion membrane, and electrolyte is also presented.

**Table 1:** Electrochemical cell parameters obtained from impedance spectroscopy measurements

WE	CE	*R*_Sol_/Ω	*R*_Carrier_/Ω	*R*_Cat_/Ω	*R*_Me_/Ω	*C*_Cat_/F	CPE-T	CPE-p
Pt	Pt	5.2 × 10	4.8 × 10^4^	—	3.1 × 10^2^	—	9.1 × 10^−5^	8.8 × 10^−1^
GC	Pt	5.2 × 10	4.5 × 10^4^	—	3.1 × 10^2^	—	5.2 × 10^−5^	8.9 × 10^−1^
(Cl-B-SubPc)/CB	Pt	5.2 × 10	2.3 × 10^4^	2.3 × 10^2^	3.1 × 10^2^	4.2 × 10^−6^	2.1 × 10^−5^	8.7 × 10^−1^
(Cl-B-SubPc)/CB	Pt	5.2 × 10	4.9 × 10^5^	1.3 × 10^2^	3.1 × 10^2^	6.2 × 10^−6^	1.1 × 10^−4^	8.2 × 10^−1^
(C-B-SubPc)/CB	Pt	5.2 × 10	1.6 × 10^6^6	3.1 × 10^2^	3.1 × 10^2^	5.3 × 10^−6^	1.3 × 10^−4^	9.4 × 10^−1^

The relatively low impedance values observed, particularly in the modified systems, indicate efficient electron and ion transport across the electrode–electrolyte interface. These results reflect the high conductivity and favorable integration of components within the electrochemical cell, further validating the suitability of Cat 1 and Cat 2 for practical CO_2_ electroreduction applications.

### Specific surface area testing (BET)

Nitrogen adsorption–desorption isotherms were recorded to evaluate the textural properties of Cat 1 and Cat 2 (Figure S9). Cat 1 (800 °C) exhibited a type I/IV isotherm characteristic of hierarchical microporous–mesoporous structures, with a BET surface area of 29.5 m^2^ g^−1^, a total pore volume of 0.10 cm^3^ g^−1^, and an average pore diameter of 10.1 nm. In contrast, Cat 2 (1000 °C) displayed a higher BET surface area of 49.3 m^2^ g^−1^ and a greater mesoporous contribution, with an average pore diameter of 11.3 nm and a total pore volume of 0.17 cm^3^ g^−1^. The enhanced mesoporosity and increased graphitization, accompanied by fewer B–N doped sites in Cat 2, facilitate improved mass transport and active site accessibility, correlating with its larger ECSA and higher CO selectivity. These findings confirm that temperature-driven structural evolution tunes the balance between micropores and mesopores, thereby influencing catalytic performance.

### Controlled potential electrolysis and product analysis

To evaluate the catalytic performance under steady-state conditions, controlled potential electrolysis (CPE) was performed over a 24-hour period at applied potentials ranging from −0.4 to −1.2 V *vs.* the reversible hydrogen electrode (RHE). The experiments were conducted at current densities ranging from 3.96 to 16.06 mA cm^−2^ for Cat 1, and 3.15 to 18.9 mA cm^−2^ for Cat 2, as shown in Fig. 3. All electrolysis experiments were carried out in a two-compartment H-type electrochemical cell separated by a Nafion membrane, allowing physical separation of the cathodic and anodic chambers to prevent product crossover. The identification and quantification of products were achieved using ^1^H NMR spectroscopy for the liquid phase and gas chromatography with a barrier ionization detector (GC-BID) for the gaseous phase (Fig. S10 and S11). For both catalysts, the primary liquid-phase products included formate, methanol, and acetate, while CO and H_2_ were the dominant gaseous species.

### Electrocatalytic performance of Cat 1

**Fig. 4:**
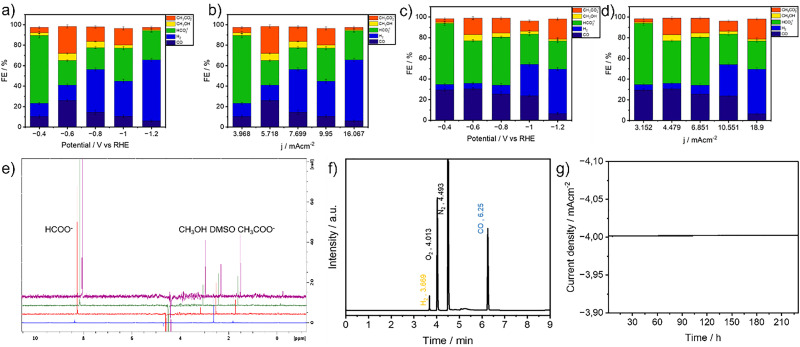
(a) Faradaic efficiencies for CO, H_2_, HCOO^−^, CH_3_OH, and CH_3_COO^−^ obtained during one-hour electrolysis at each potential displayed. (b) Faradaic efficiencies for CO, H_2_, HCOO^−^, CH_3_OH, and CH_3_COO^−^ obtained during one-hour electrolysis at each current density displayed (Cat 1, Cl-B-SubPc)/CB (pyrolyzed at 800 °C). (c) Faradaic efficiencies for CO, H_2_, HCOO^−^, CH_3_OH, and CH_3_COO^−^ obtained during one-hour electrolysis at each potential displayed. (d) Faradaic efficiencies for CO, H_2_, HCOO^−^, CH_3_OH, and CH_3_COO^−^ obtained during one hour electrolysis at each current density displayed (Cat 2, Cl-B-SubPc)/CB (1000 °C). (e) NMR spectra recorded after 1 hour, 3 hours, 6 hours and 24 hours of electrocatalysis, at −0.4 V *vs.* RHE (Cat 2, Cl-B-SubPc)/CB (1000 °C). (f) GC-BID-chromatogram gas products formed after CO_2_ reduction at −0.4 V *vs.* RHE for 1 h by (Cat 2, Cl-B-SubPc)/CB (1000 °C) catalyst modified carbon paper electrode in 0.1 M CO_2_-saturated CsHCO_3_ solution. (g) Long-term electrolysis experiment of Cat 1/CB at −0.4 V *vs.* RHE of total liquid products.

The electrochemical behavior of Cat 1 immobilized on carbon paper was assessed by tracking the faradaic efficiencies (FEs) of key products at various applied potentials (Fig. 4(a), (b) and Fig. S12). At −0.4 V *vs.* RHE, Cat 1 exhibited high selectivity toward formate, achieving an FE of 66.2%, along with 5.2% for acetate and 2.8% for methanol. This suggests that low overpotentials promote selective two-electron reduction pathways conducive to formate formation.

As the applied potential increased to −0.6 V, the selectivity began to shift. The FE for methanol and acetate increased, reaching 6.9% and 26.42%, respectively, while CO production maintained a high FE of 26.01%. At −0.8 V, CO production peaked with a faradaic efficiency of 26.92%, after which further increases in overpotential led to a gradual decline, likely due to the increasing dominance of the hydrogen evolution reaction (HER).

### Electrocatalytic performance of Cat 2

The performance of Cat 2 supported on carbon paper was similarly assessed under identical electrolysis conditions (Fig. 4(c), (d) and Fig. S13). The catalyst produced the same primary products CO, H_2_, formate, methanol, and acetate but with notable differences in selectivity trends. Acetate production showed a nonlinear relationship with applied potential: the FE reached 16.3% at −0.6 V, decreased to 9.9% at −1.0 V, and increased again to a maximum of 19.49% at −1.2 V. CO selectivity followed a typical trend, increasing at moderate potentials and declining at more negative ones due to intensified HER. The FE for methanol reached 6.9% at −0.6 V, then decreased to 3.2% at −1.0 V, reflecting a potential-dependent shift in product distribution. Long-term electrolysis at fixed potentials showed stable current densities and product distributions over 24 hours (Fig. 4(e)). These findings indicate that the Cat 2/CB catalyst enables efficient and selective CO_2_ electroreduction to CO, methanol, and acetate, with suppressed HER activity and sustained electrochemical performance under operational conditions.

Although Cat 2 showed a higher CO selectivity than Cat 1 across most potentials, it exhibited slightly lower FEs for methanol and acetate, suggesting differences in surface interaction or intermediate stabilization mechanisms between the two catalysts.

The operational stability of the Cat 1/CB catalyst was systematically evaluated through prolonged NMR testing conducted at an applied potential of −1.0 V *vs.* RHE. Over a continuous 220-hours electrolysis period, the catalyst demonstrated remarkable durability (Fig. 4(g) and Fig. S14), as evidenced by the minimal fluctuation in both current density and faradaic efficiency (FE) for liquid-phase products (Fig. 4(a)–(d)). Although a slight decline in FE was observed from 75.43% at the beginning to 71.3% at the end of the test the overall performance remained stable, with only marginal losses. This small reduction suggests minor deactivation or changes in the electrode–electrolyte interface over time, yet the system retained most of its electrocatalytic activity throughout the extended operation. Such stability highlights the robust interaction between the catalyst layer and the carbon support, as well as the resilience of the catalyst under continuous electrochemical stress. Nonetheless, the observed decline albeit modest indicates potential opportunities for further engineering of the catalyst architecture or electrolyzer configuration to mitigate degradation mechanisms and ensure sustained high efficiency in practical, long-term CO_2_ reduction applications. To ascertain the true origin of the carbonaceous products and eliminate the possibility of contributions from background contaminants or electrolyte decomposition, a control experiment was carried out under identical electrochemical conditions but in an argon-purged electrolyte (*i.e.*, in the absence of CO_2_). As expected, no detectable formation of hydrocarbons or oxygenated carbon products were observed in this case (Fig. S15). This finding clearly confirms that the carbon-based products namely CO, formate, methanol, and acetate obtained during CO_2_ electroreduction arise solely from the reduction of gaseous CO_2_, and not from extraneous carbon sources or degradation of the electrolyte components.

### Geometry optimization and mechanistic insights into CO_2_ reduction on B–NC@Graphite

This section presents a qualitative mechanistic framework inspired by prior literature on N-doped carbons and B, N-doped carbons.^20–22^ Earlier electrochemical studies on chlorine-bound boron subphthalocyanine demonstrated that the chlorine atom dissociates upon the first reduction, producing a radical anion with its LUMO occupied (Fig. S16a and b).^23^ By analogy, during pyrolysis, chlorine elimination yields a boron–N-doped graphitic lattice (B–NC@graphite) (Fig. 2(c) and (g)). Substitutional boron–nitrogen sites introduce acceptor-donor-like defect states above the valence band maximum, modifying the local density of states (LDOS) and shifting the Fermi level *E*_F_. This breaks electron–hole symmetry in the π-electron system of graphite, enhancing local charge accumulation, localized most probably on the nitrogen atoms and reactivity toward CO_2_ adsorption (Fig. S15a). Carbon monoxide pathway: at the boron–N-doped site, electron injection enhances the local charge density. This excess electron stabilizes adsorbed CO_2_ by partial charge transfer, bending the molecule from its linear configuration. Proton-coupled electron transfer (PCET) processes sequentially reduce the adsorbate, and the resulting orbital overlap facilitates C–O bond cleavage. The reaction yields CO + H_2_O (Fig. 4(f) and Fig. S17a), while the electronic occupation of the boron–N site relaxes back to its ground-state LDOS profile. Methanol pathway: under higher overpotential, additional electronic states near *E*_F_ are populated, allowing the same boron–N-site to stabilize multi-electron intermediates. The reaction coordinate can be represented as a stepwise sequence of PCET transitions. The B–N-induced LDOS perturbation shifts the Fermi level alignment, favoring charge delocalization into antibonding orbitals of CO_2_-derived intermediates. NMR spectra confirm methanol formation *via* a ^1^H-NMR resonance at 3.32 ppm (Fig. 4(e) and Fig. S17b). Formate pathway: for formate, the catalytic cycle begins with a single-electron transfer into a surface-bound CO_2_ molecule (CO_2_˙^−^) localized at the boron–N-site. Protonation yields the formate intermediate (HCOO^−^), which desorbs upon completion of electron transfer (Fig. S18). The boron–N site is re-equilibrated to its conductive ground state, ensuring catalytic turnover. Acetate pathway: as already described above, adjacent nitrogen atoms within B–N sites provide a platform for CO coupling. Two CO molecules within the same B–N site, stabilized through surface charge redistribution, undergo dimerization. The conductive graphitic matrix ensures that the electronic overlap between the CO π* orbitals and the perturbed LDOS remains sufficient for bond formation. Protonation and electron transfer then reduce the intermediate to glyoxal, hydroxyacetaldehyde, and finally acetic acid. In alkaline solution, acetic acid deprotonates to acetate, detected at 1.87 ppm (Fig. 4(e)). The cycle remains stable because the boron–N site electronic structure is periodically restored by re-equilibration with the extended π-band.

### Zero-gap cell electrolyzer test

To assess the scalability and technological relevance of the catalytic system beyond conventional H-cell testing, electrochemical CO_2_ reduction was further evaluated in a zero-gap flow cell electrolyzer. This architecture has emerged as a promising alternative in recent years, as it minimizes the separation between the gas diffusion electrode (GDE) and the ion-conducting membrane, thereby removing the bulk liquid electrolyte layer characteristic of H-type cells. Such a configuration effectively reduces ohmic resistance, improves ionic conductivity, and facilitates rapid CO_2_ transport to active sites, which collectively enables operation at higher current densities with greater energy efficiency.^24,25^ While these attributes mark a clear advantage over H-cell and liquid flow cell designs, zero-gap electrolyzers remain inherently limited to the generation of gaseous products, constraining the broader product scope accessible through CO_2_ electroreduction.

**Fig. 5:**
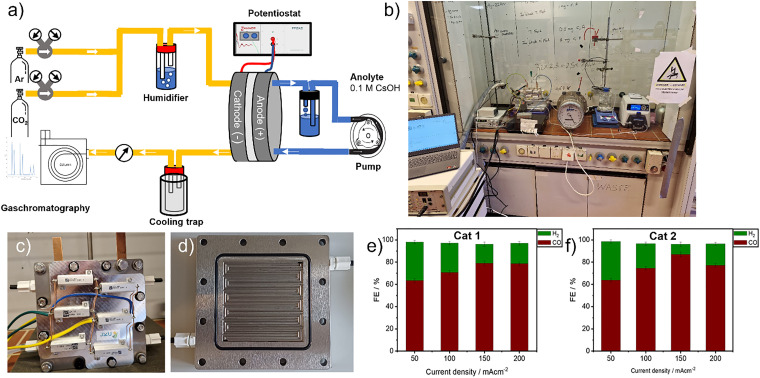
(a)–(d) Schematic illustration and photograph of a laboratory-scale zero-gap electrolyzer. (e) The CO_2_ reduction performance of (Cat 1, Cl-B-SubPc)/CB (800 °C) at 50, 100, 150, and 200 mA cm^−2^ at 60 °C after two hours of electrolysis. All investigated GDEs possessed a catalytic loading of 0.5 mg cm^−2^ of active material. (f) The CO_2_ reduction performance of (Cat 2, Cl-B-SubPc)/CB (1000 °C at 50, 100, 150, and 200 mA cm^−2^ at 60 °C) after two hours of electrolysis. All GDEs examined contained a catalytic loading of 0.5 mg cm^−2^ of active material.

A custom-built zero-gap electrolyzer was constructed for this study (Fig. 5(a)–(d) and Fig. S19). The device consisted of stainless steel flow plates, a catalyst-coated working electrode, an IrO_2_-based anode as the counter electrode, chemically resistant Teflon spacers, and a high-performance anion exchange membrane (PiperION A40, 40 μm thickness). The PiperION membrane was selected for its high hydroxide ion conductivity and robust mechanical stability, which are essential for long-term electrolysis operations under alkaline conditions.^26,27^

The integration of CO_2_ capture and conversion technologies into a single reactor platform is increasingly considered critical for industrial CO_2_ reduction applications. Traditional systems rely on external CO_2_ gas supplies, which add significant energy and economic costs. Therefore, the use of electrolyzers compatible with direct CO_2_ feedstocks such as flue gas or bicarbonate-rich solutions is gaining interest as a means to streamline the carbon capture-utilization cycle.^28–30^

In the current setup, the cathode comprised a gas diffusion electrode (GDE) loaded with the synthesized catalyst at a geometric loading of 1 mg cm^−2^. A PiperION A40-HCO_3_ anion exchange membrane was used to separate the cathode and anode compartments. Prior to assembly, the membrane was conditioned in 1 M KOH for 12 hours to fully activate the exchange sites, followed by rinsing with Milli-Q water to eliminate residual electrolyte and avoid ion contamination. The anode was a commercial IrO_2_-coated electrode (loading: 1 mg cm^−2^), selected for its superior stability and oxygen evolution reaction (OER) performance in alkaline media.^31^

The electrolyte employed on the anode side was a 0.1 M aqueous cesium hydroxide (CsOH) solution. Cesium-based electrolytes are often used in CO_2_ electroreduction studies due to their relatively large ionic radius and ability to promote CO_2_ adsorption while stabilizing key reaction intermediates.^32^ Meanwhile, high-purity CO_2_ gas (99.99%) was continuously introduced at a flow rate of 50 mL min^−1^ behind the GDE cathode, enabling effective gas-phase diffusion into the catalyst layer and subsequent electrochemical conversion.

This zero-gap configuration allowed for tight control over electrolysis conditions, including cell temperature, electrolyte composition, and gas flow, making it highly suitable for investigating performance metrics such as faradaic efficiency, current density, and product selectivity. Additionally, this design offers a promising platform for future scale-up and integration with carbon capture systems, including direct air capture (DAC) or post-combustion CO_2_ sources.^33,34^

The CO_2_ feed was humidified *via* a temperature-controlled humidifier, with the relative humidity dynamically adjusted in accordance with the applied current density to optimize reaction conditions. Fresh electrolyte solution was prepared prior to each experimental run and continuously circulated through the cell at a flow rate of 50 mL min^−1^ using peristaltic pumps. Under zero-gap cell conditions, the optimized parameters including a current density of 150 mA cm^−2^, a high gas flow rate of 50 mL min^−1^, operation at 60 °C, and a low CsOH concentration of 0.1 M resulted in maximum faradaic efficiencies (FE) of 79.03% for Cat 1 and 87.38% for Cat 2 (Fig. 5(e) and (f)). These results highlight the potential of carefully engineered CsOH-based electrolyzers for efficient, integrated CO_2_ capture and electrochemical conversion. The much higher FE_CO_ observed in the zero-gap electrolyzer (up to 87%) compared to the H-cell (∼27%) originates from the differences in mass transport and ohmic resistance. In H-cells, CO_2_ solubility in aqueous electrolyte (*ca.* 33 mM) limits availability at the electrode, while in zero-gap configuration, continuous gas feed directly contacts the GDE, minimizing transport limitations and favoring CO production.

## Conclusions

This work introduces two boron NC@ graphite catalysts as promising noble-metal-free electrocatalysts for the selective electrochemical reduction of CO_2_ to C_1_ and C_2_ products. Utilizing both H-cell and zero-gap electrolyzer configurations, the catalysts demonstrated the formation of valuable gas-phase products (CO, H_2_) and liquid-phase products (formate, methanol, acetate), verified through detailed spectroscopic and electrochemical analyses. Notably, in a zero-gap electrolyzer setup, Cat 1 achieved a faradaic efficiency (FE) of 79.03% for CO at a current density of 150 mA cm^−2^, while Cat 2 reached a remarkable FE of 87.38% under similar conditions. These results highlight the high selectivity and catalytic performance of the B-subphthalocyanine systems under industrially relevant operating parameters. Overall, this study establishes boron-based subphthalocyanines as a new class of highly efficient, metal-free molecular electrocatalysts for CO_2_ conversion. The findings offer valuable mechanistic insights and lay the groundwork for developing scalable, sustainable strategies in carbon capture, utilization, and renewable energy storage.

## Author contributions

Conceptualization: WS. Formal analysis: FY, SO, SV, DK. Funding acquisition: WS. Investigation FY, SO, SV. Methodology FY, DK, VS, SO, WS. Project administration: WS. Resources: WS. Supervision: WS. Validation: FY, DK, WS. Visualization: FY, WS. Writing – original draft: FY, WS. Writing – review & editing FY, DK, WS.

## Conflicts of interest

There are no conflicts to declare.

## Supplementary Material

YA-004-D5YA00260E-s001

## Data Availability

The datasets supporting this article have been uploaded as part of the supplementary information. Further information not provided can be obtained upon request by the authors. Supplementary information is available. See DOI: https://doi.org/10.1039/d5ya00260e.
