# Supplementary material for: Efficient electroreduction of CO2 to C1 and C2 products using atomically dispersed boron N–C@graphite catalysts
Source: Energy Adv. 2025 Oct 22;4(12):1443–54. doi: 10.1039/d5ya00260e (PMC12560087; doi:10.1039/d5ya00260e)
Supplement: YA-004-D5YA00260E-s001 [file YA-004-D5YA00260E-s001.pdf]

Supplementary Information (SI) for Energy Advances.
This journal is © The Royal Society of Chemistry 2025

Supplementary Informations

Efficient Electroreduction of CO_2 to C1 and C_2 Products Using Atomically Dispersed Boron N-C@Graphite Catalysts

Farzaneh Yari, Simon Offenthaler, Sankit Vala, Dominik Krisch, and Wolfgang Schöfberger*

Farzaneh Yari, Simon Offenthaler, Sankit Vala, Dominik Krisch, Prof. Dr. W. Schöfberger
Institute of Organic Chemistry, Laboratory for Sustainable Chemistry and Catalysis (LSusCat)
Johannes Kepler University (JKU)
Altenberger Straße 69, 4040 Linz, Austria.
E-mail: wolfgang.schoefberger@jku.at
Homepage: https://www.jku.at/en/institute-of-organic-chemistry/team/schoefberger-lab

Supporting information for this article is given via a link at the end of the document.

Keywords: Keywords: CO_2 electrocatalysis • boron subphthalocyanine • pyrolysis • boron-NC@graphite • H-cell electrolyzer • zero-gap cell electrolyzer

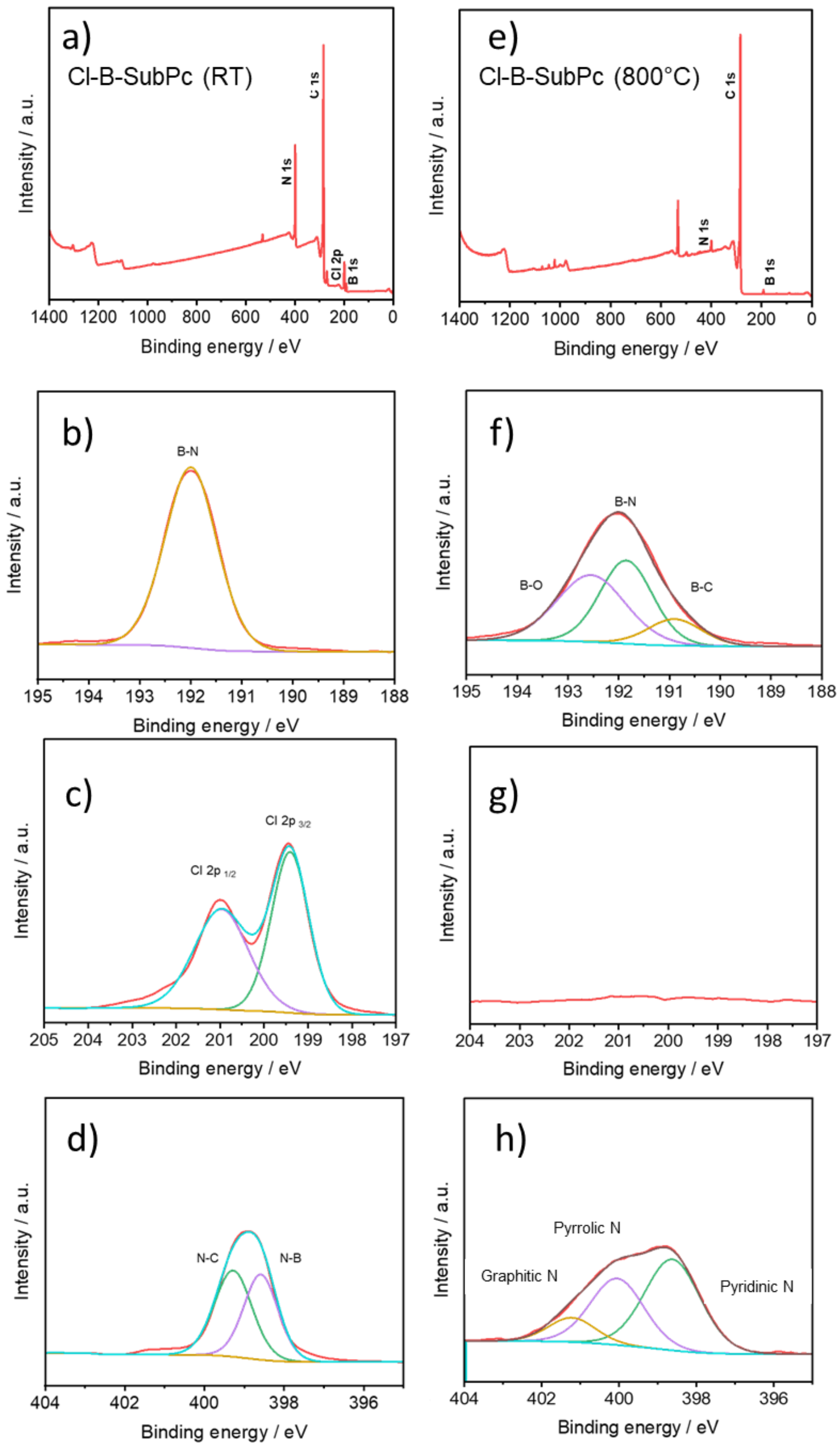

Figure S1. XPS spectra of the molecular Cl-B-SubPc a) survey energy scan b) B 1s, c) Cl 2p d) N1s, and XPS spectra of Cl-B-SubPc after controlled pyrolysis at 800°C e) survey energy scan f) B 1s, g) Cl 2p h) N1s.

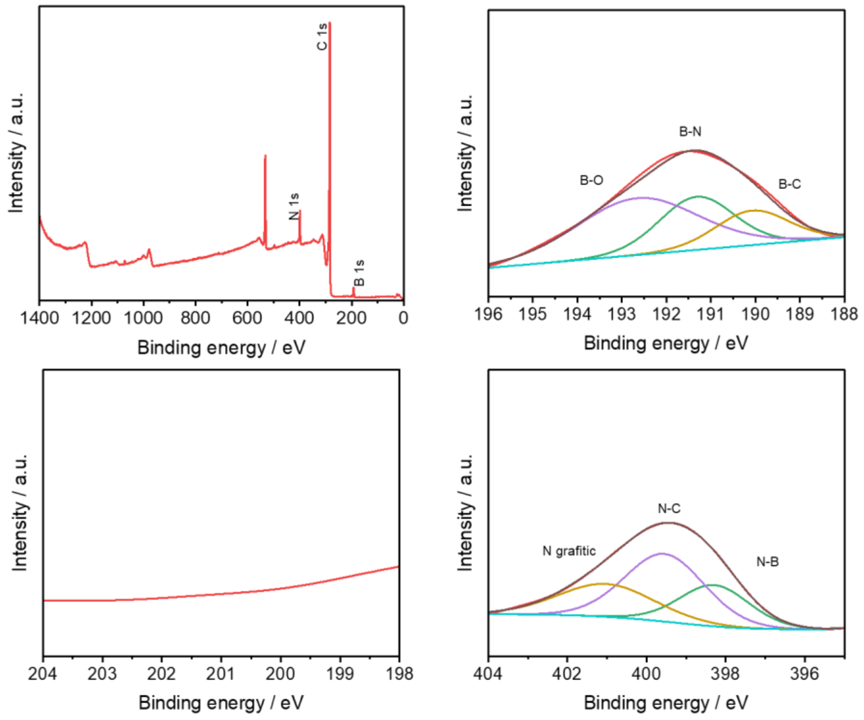

Figure S2. XPS spectra of the (Cl-B-SubPc)/CB (1000°C) after 24 hours of electrolysis at -1.0 vs RHE a) full-energy scan, b) B 1s, c) Cl 2p and b) N1s.

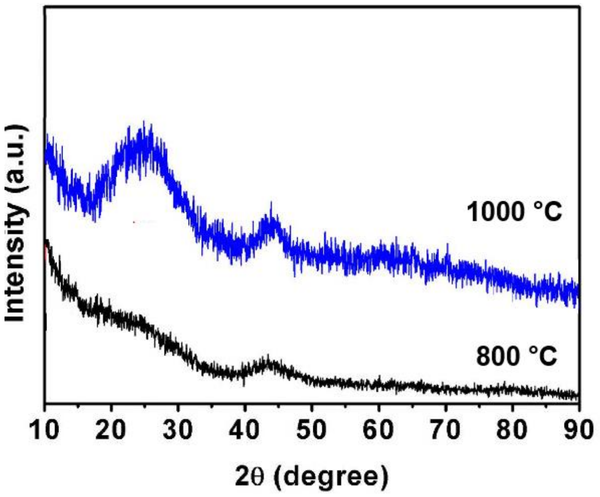

Figure S3. XRD measurements of **Cat 1** (800°C) and **Cat 2** (1000°C)

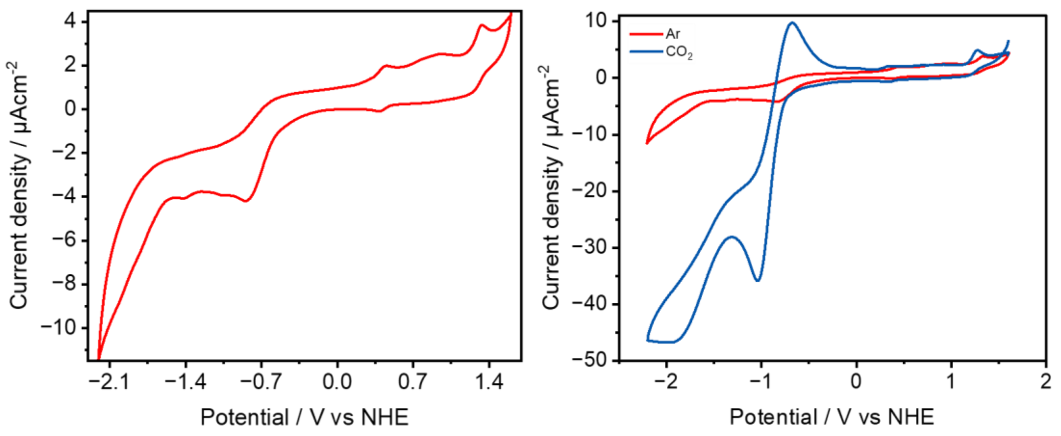

Figure S4. Comparison of cyclic voltammograms of (Cl-B-SubPc) dissolved in DCM under argon and CO_2 containing 0.1 M TBAP as supporting electrolyte with glassy carbon as working, platinum wire as counter and nonaqueous pseudo-Ag/AgCl as reference electrode with a scan rate of 30 mVs^{-1}.

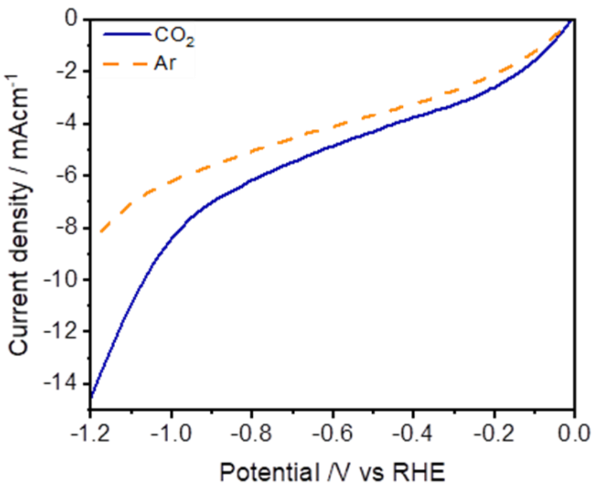

Figure S5. LSV curves at a scan rate of 30 mV s^{-1} (Cl-B-SubPc)/CB (800°C) /CB WEs with Ar and CO_2 saturation 0.1M $CsHCO_3$

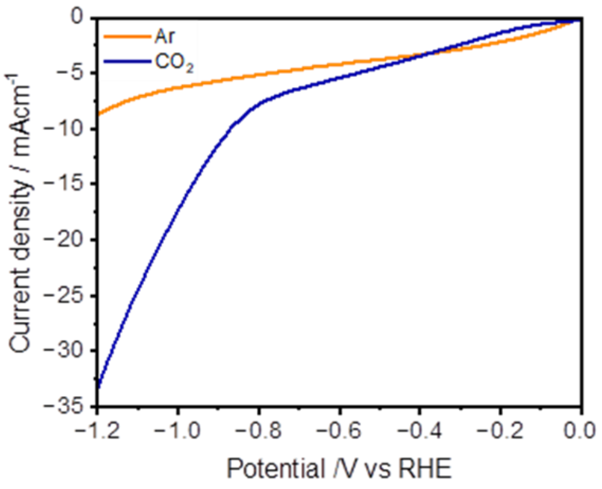

Figure S6. LSV curves at a scan rate of 30 mV s^{-1} (Cl-B-SubPc)/CB (1000°C) WEs with Ar and CO_2 saturation 0.1M $CsHCO_3$

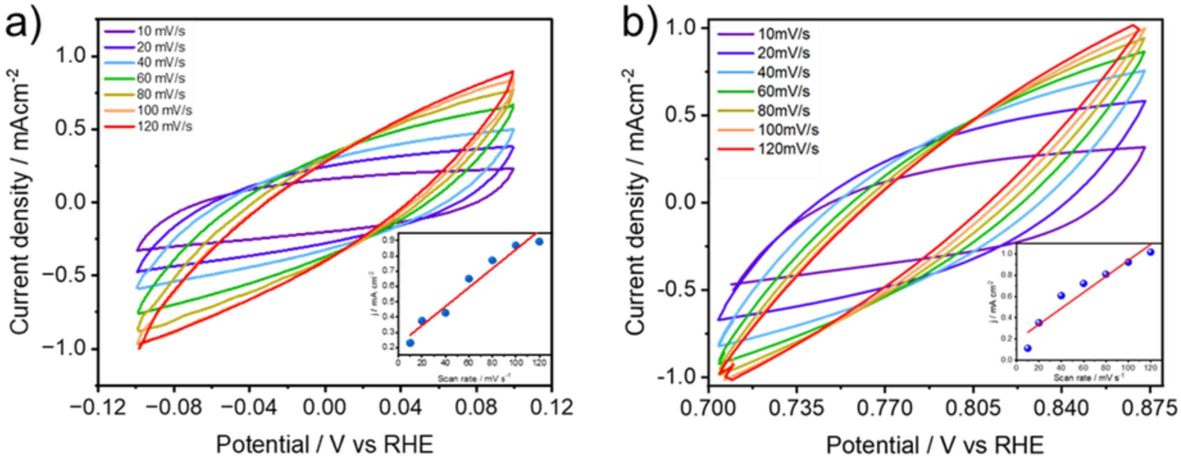

Figure S7. a) Cyclic voltammograms of (Cl-B-SubPc)/CB (800°C) at different sweep rates of 10, 20, 40, 60, 80, 100, and 120 mV s^{-1} in 0.1M $CsHCO_3$ and a linear plot of capacitive current versus scan rate. b) Cyclic voltammograms of (Cl-B-SubPc)/CB (1000°C) at different sweep rates of 10, 20, 40, 60, 80, 100, and 120 mV s^{-1} in 0.1M $CsHCO_3$ and a linear plot of capacitive current versus scan rate.

Characterization by Electrochemical Impedance Spectroscopy (EIS)- Method

The electrochemical impedance spectroscopy (EIS) was measured using an IVIUM CompactStat (Netherlands). The impedance spectrum was recorded for all experiments in the frequency range of 10^5 Hz to 0.01 Hz with a perturbation amplitude of 10 mV. The aim of this characterization is the investigation of **Cat 1** and **Cat 2** for the carbon dioxide reduction electrolysis, first two platinum electrodes were measured in a one-cell compartment with the corresponding electrolyte as a control experiment to determine the electrolyte resistance. Subsequently, the setup was transitioned to an H-cell configuration with a Nafion membrane, enabling the determination and subtraction of the membrane resistance from the electrolyte resistance. Further experiments involved replacing one platinum electrode with a carbon paper electrode as the working electrode. Lastly, the carbon paper, coated with **Cat 1** and **Cat 2**, served as the working electrode for the complete electrochemical cell evaluation through EIS. The resulting fitted and calculated impedance data, as well as resistance values for each cell component.

The Bode plot for the two-electrode system is also shown in Figure S11. All resistance values for each cell component, i.e., electrolyte solution, membrane, and carrier electrode are shown in Table S1 for carbon dioxide reduction cell systems, respectively. Based on EIS, the applied electrochemical cells were characterized in detail indicating negligible losses of the systems.

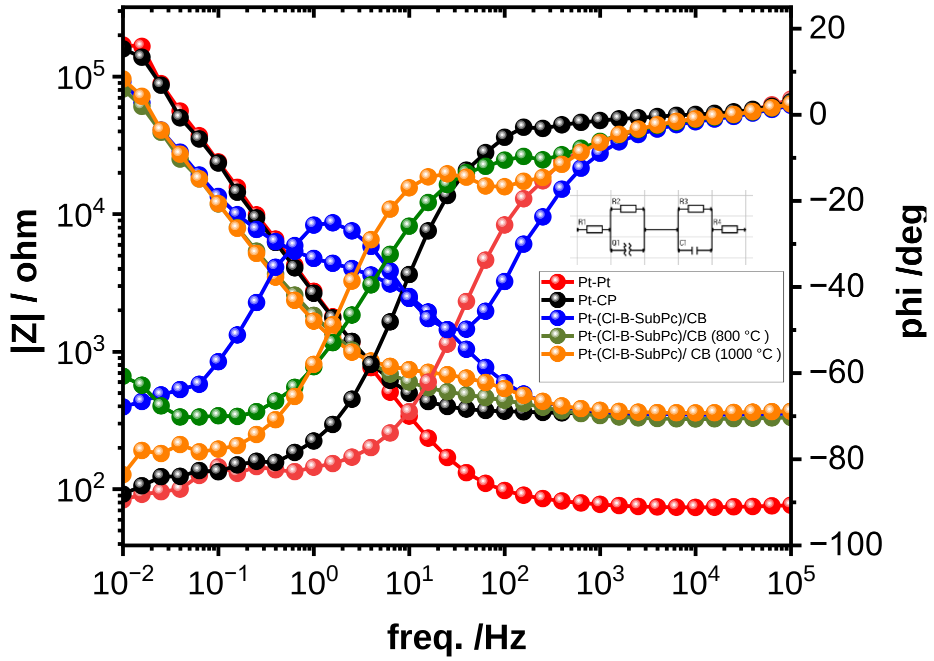

Figure S8. Bode plot recorded via electrochemical impedance spectroscopy of (Cl-B-SubPc) **1**/CB and (Cl-B-SubPc-$OC_{12}H_{23}$) **2**/CB catalyst modified carbon paper electrode in the frequency range of $1*10^{-1}$ Hz to $1*10^{5}$ Hz with a perturbation amplitude of 10 mV.

Table S1 Cell parameters extracted via electrochemical impedance measurements.

WE	CE	R_{Sol}/Ω	$R_{Carrier}/\Omega$	R_{Cat}/Ω	R_{Me}/Ω	C_{Cat}/F	CPE-T	CPE-p
Pt	Pt	5.2E+01	4.8E+04	-	3.1E+02	-	9.1E-05	8.8E-01
GC	Pt	5.2E+01	4.5E+04	-	3.1E+02	-	5.2E-05	8.9E-01
(Cl-B-SubPc)/CB	Pt	5.2E+01	2.3E+04	2.3E+02	3.1E+02	4.2E-06	2.1E-05	8.7E-01
(Cl-B-SubPc)/CB	Pt	5.2E+01	4.9E+05	1.3E+02	3.1E+02	6.2E-06	1.1E-04	8.2E-01

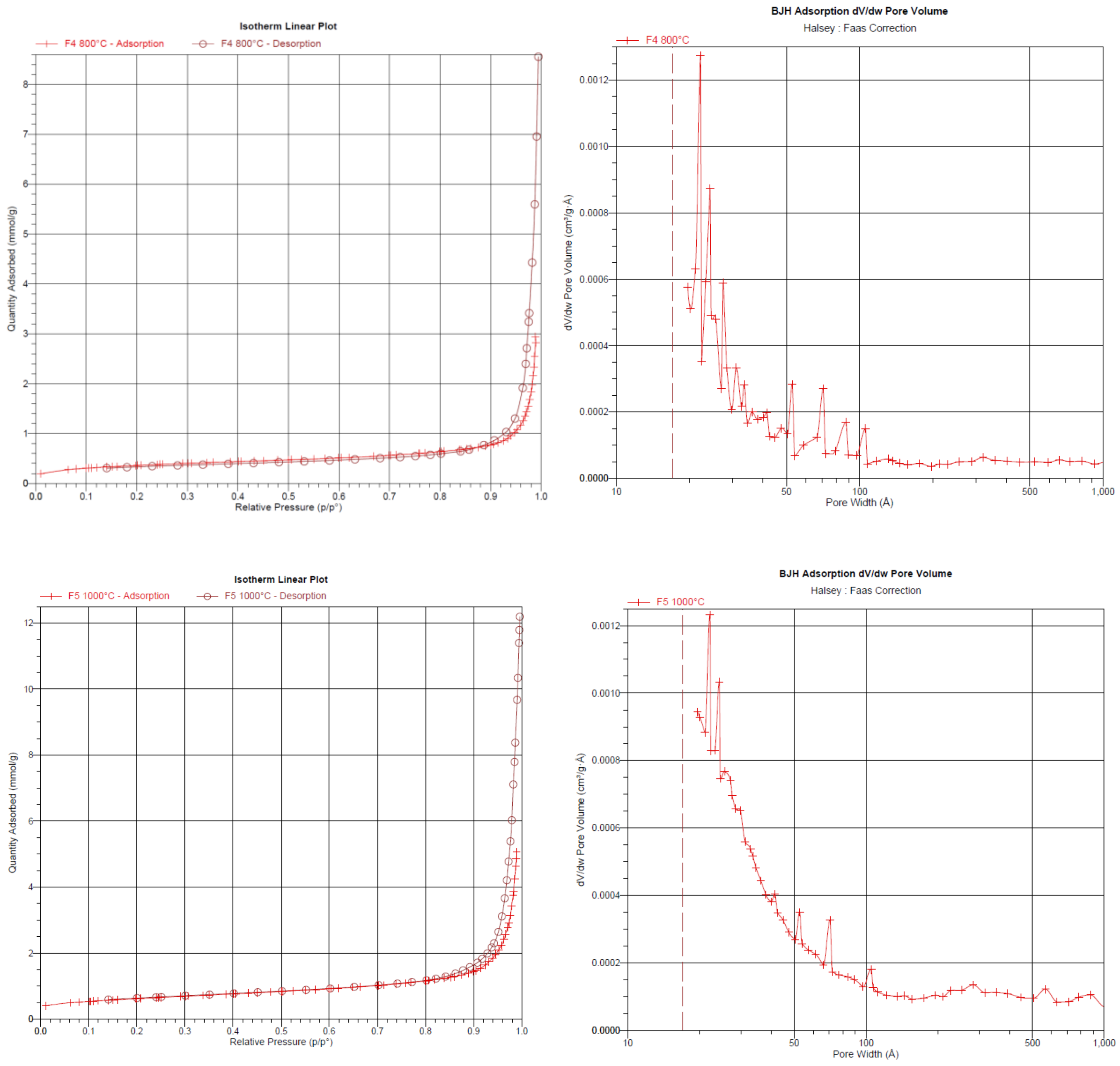

Figure S9. N_2 adsorption–desorption isotherms of **Cat 1** (top, 800 °C) and **Cat 2** (bottom, 1000 °C) and the respective pore size distribution curves of **Cat 1** and **Cat 2** obtained from BJH analysis.

Product detection and quantification by ^{1}H-NMR

All liquid products have been quantified by a Bruker Avance III 300 MHz NMR spectrometer and a Bruker Avance III 500 MHz NMR spectrometer using D_2O as solvent. 50 μL of DMSO solution was used as internal standard. For this purpose, we have followed a previously reported procedure.[1] 450 μL analyte was taken directly from the reaction mixture for the ^{1}H-NMR analysis. Further suppression of the water peak was conducted in order to make the CO_2-reduced product peaks visible. Formate was found as only reduced product from ^{1}H-NMR with corresponding peaks at 8.4 ppm. Number of scans and other spectral acquisition parameters were kept fixed during all acquisitions. During the quantification of the samples, each peak was normalized with respect to the DMSO peak at 2.7 ppm (Figure S8).

The Faradaic efficiency (*FE*, %) of the liquid products can be quantified according to this formula,

$$FE_i = \frac{z_i \cdot n_i \cdot F}{Q_{\text{total}}} = \frac{z_i \cdot \frac{V}{V_{\text{test}}} \cdot y_i}{Q_{\text{total}}} \cdot F$$

where y_i, V and V_{test} are the mole amount of liquid product directly measured by ^{1}H NMR spectra, the volume of total electrolyte (30 mL) and the volume of the tested electrolyte (0.2 mL), respectively.

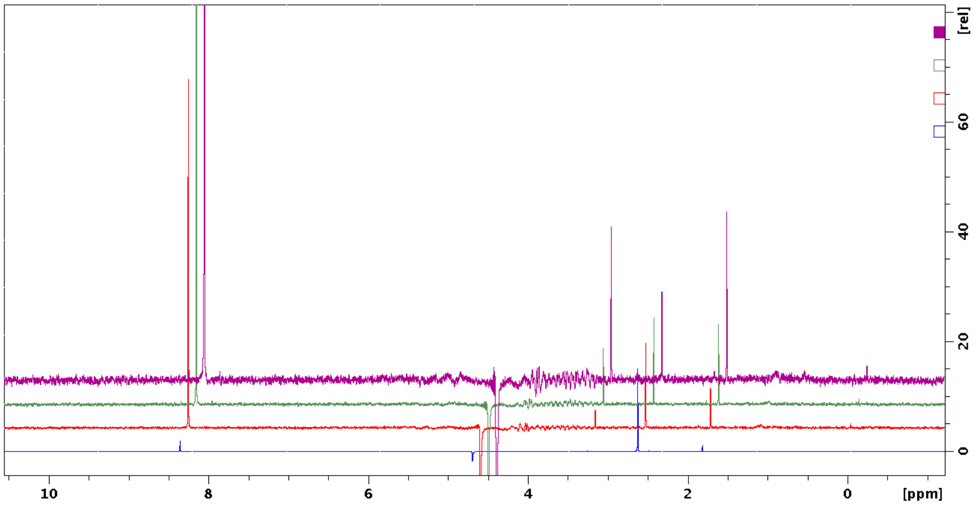

Figure S10. Time-dependent ^{1}H-NMR spectra of the liquid products formed after CO_2 reduction at -0.4 V vs RHE by (Cl-B-SubPc)/CB 1000°C pyrolyzed catalyst-modified efficiency Carbon Paper electrode in 0.1M CO_2 -saturated $CsHCO_3$ solution.

Product analysis by GC-BID

Product analysis, conducted via Gas-Chromatograph Nexis GC-2030 by Shimadzu. During the electrolysis in the closed cell, 250 µL of the headspace gas was taken for the quantification of evolved gas. CO and H_2 were detected during the electrolysis in the cathodic region (Figure S12). For quantification purposes, the peak area was converted into a gas volume using the calibration curve.

The Faradaic efficiency (FE%) of the gas products can be quantified following this formula,

$$FE_i = \frac{z_i \cdot n_i \cdot F}{Q_{\text{total}}} = \frac{z_i \cdot \frac{f_{CO_2} \cdot t}{V_{\text{loop}}} \cdot x_i}{Q_{\text{total}}} \cdot F$$

where z_i, n_i, Q_{total}, f_{CO2}, t, V_{loop}, x_i, and F are the number of electrons involved in the reaction, the mole amount of the total product (mol), the total consumed charge (C), the flow rate of CO_2 (5 mL min^{-1}), the test time (60 min), the volume of the quantitative loop in GC instrument used for detection (0.25 mL), the mole amount of the product directly measured by GC instrument (mol) and the faradic constant (96485.33 C mol^{-1}), respectively.

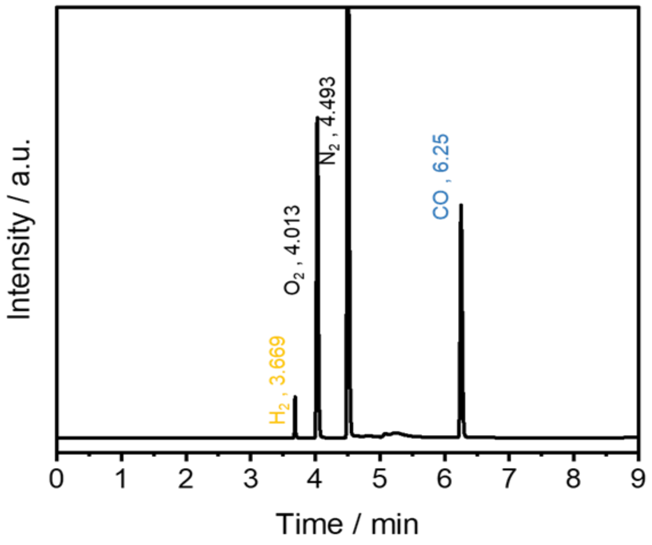

Figure S11. GC-BID-chromatogram gas products formed after CO_2 reduction at -0.6 V vs RHE for 1 h by (Cl-B-SubPc)/CB pyrolyzed at 1000°C catalyst modified carbon paper electrode in 0.1 M CO_2 -saturated $CsHCO_3$ solution.

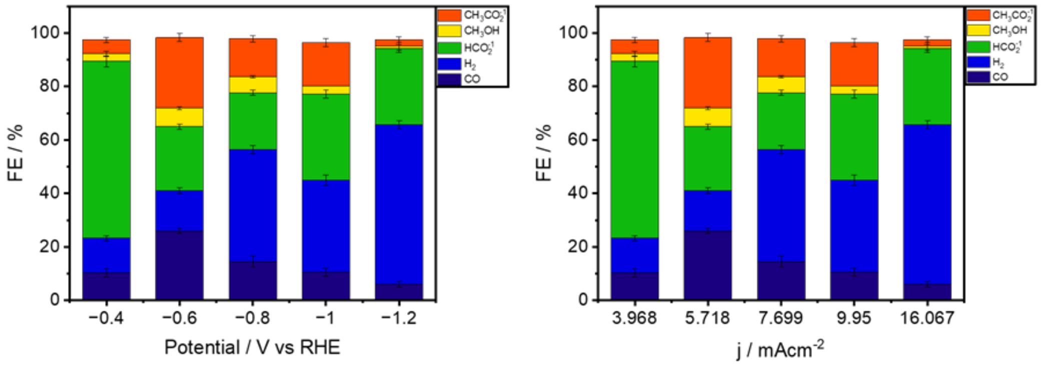

Figure S12. A) Faradaic efficiencies for CO, H_2, $HCOO^-$, CH_3OH, and CH_3COO- obtained during one-hour electrolysis at each potential displayed. B) Faradaic efficiencies for CO, H_2, $HCOO^-$, CH_3OH, and CH_3COO^- obtained during one-hour electrolysis at each current density displayed. (Cl-B-SubPc)/CB (800°C).

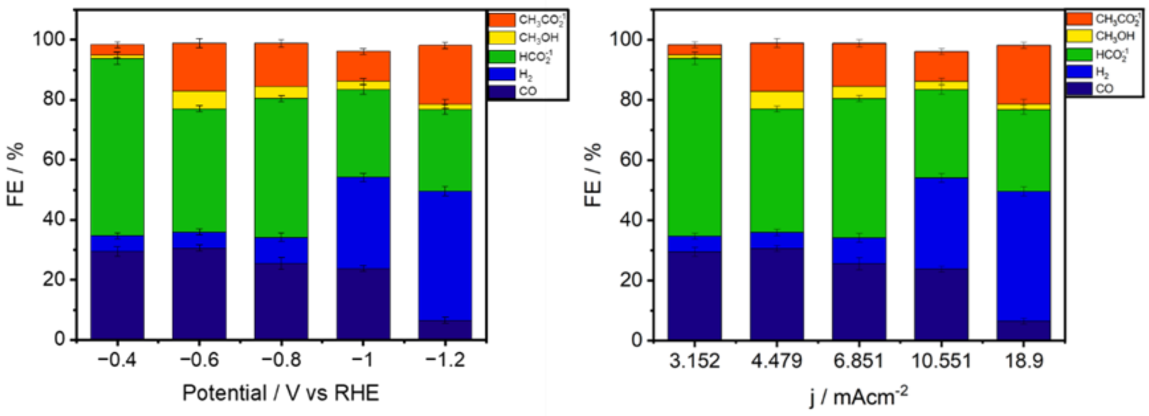

Figure S13. Faradaic efficiencies for CO, H_2, $HCOO^-$, CH_3OH, and CH_3COO^- obtained during one-hour electrolysis at each potential displayed. B) Faradaic efficiencies for CO, H_2, $HCOO^-$, CH_3OH, and CH_3COO^- obtained during one-hour electrolysis at each current density displayed. (Cl-B-SubPc)/CB (1000°C).

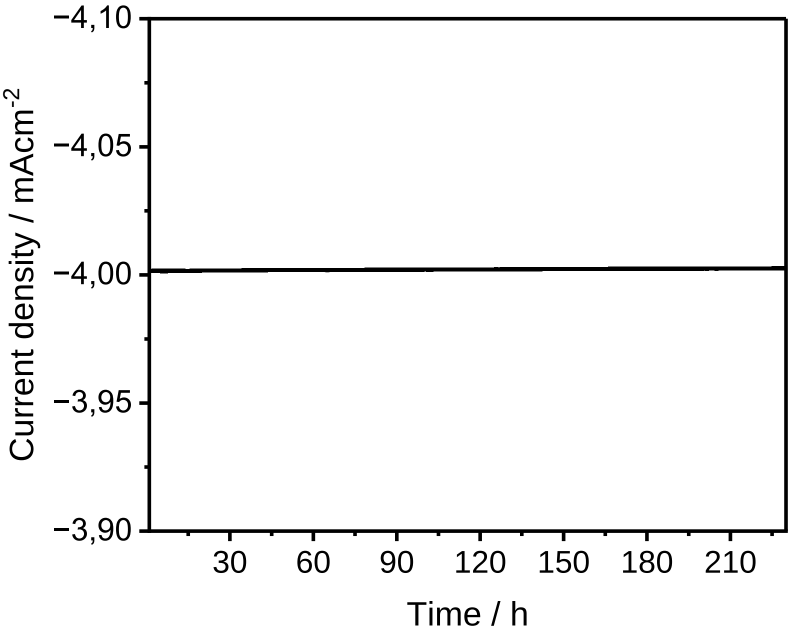

Figure S14. Long-term electrolysis experiment of the (Cl-B-SubPc)/CB in the ZGC.

TON TOF calculations

Turn Over Frequency Calculations:

Turn Over Frequency (TOF) was calculated using the equation;

$$\mathrm{TOF} = \frac{iE_F}{NFn_{cat}}$$

Where i = current

E_F = Faradaic efficiency for ethanol

N = Number of electrons in the half-reaction (N = 2 for CO_2 to CO conversion)

F = Faraday constant

n_{cat} = total moles of the catalyst employed for the electrolysis

Turn Over Number Calculations:

Turn Over Number was calculated using the equation;

$$\mathrm{TON} = \mathrm{TOF} \times t$$

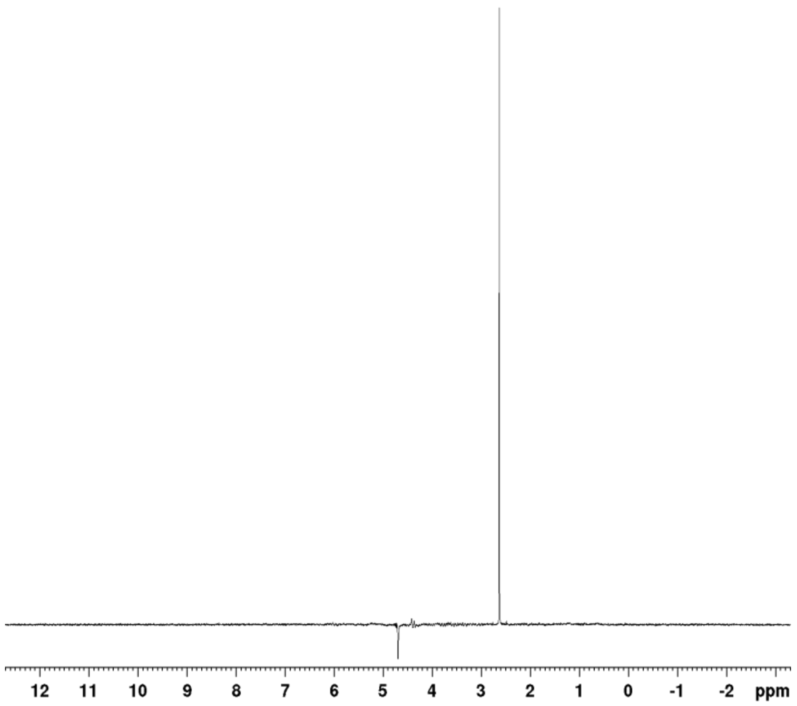

Figure S15. ^{1}H NMR spectrum Control electrolysis performed in an Ar-saturated electrolyte**.**

Geometry Optimization, Mechanistic Insights

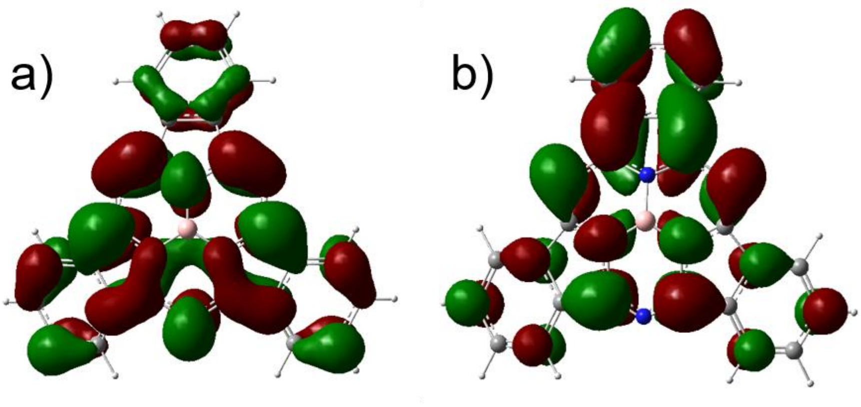

Figure S16. a) HOMO and b) LUMO of B-SubPc anion radical.

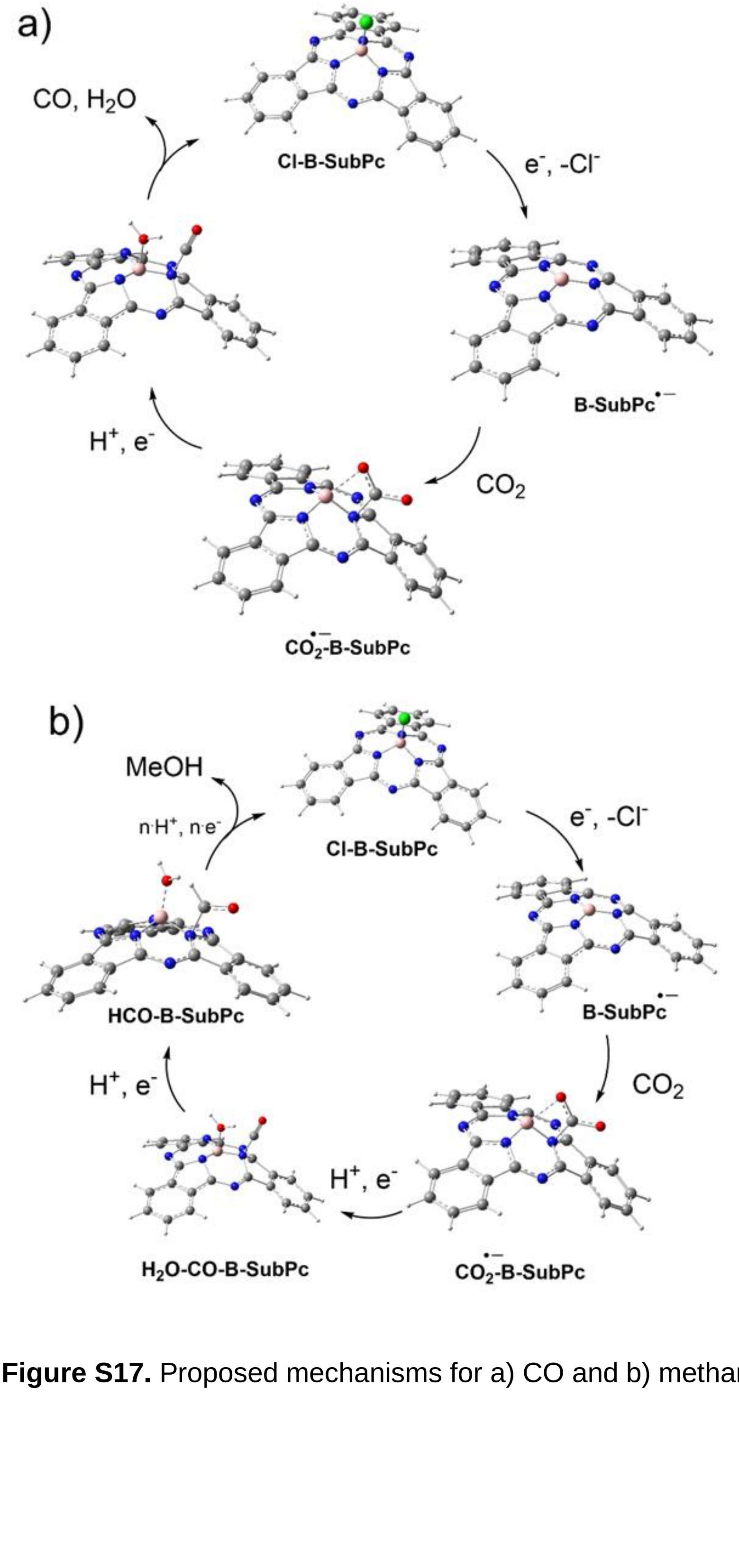

Figure S17. Proposed mechanisms for a) CO and b) methanol production.

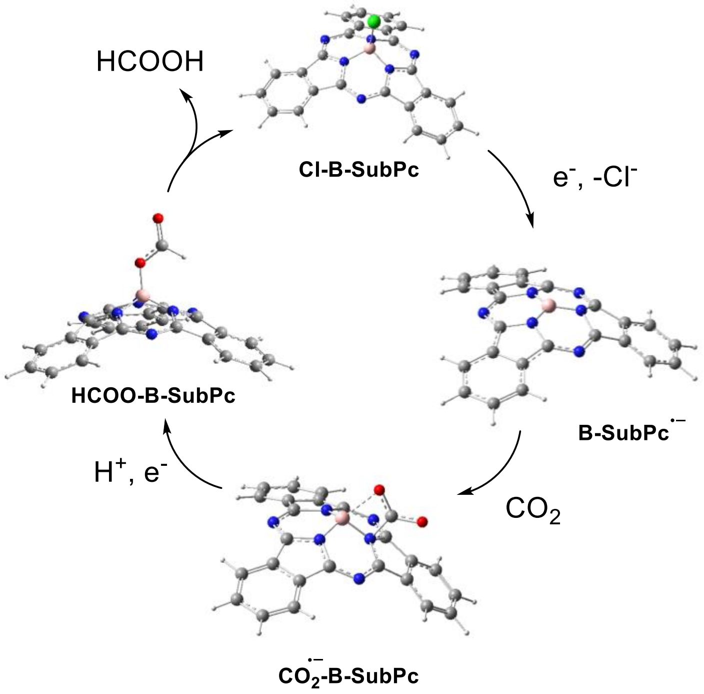

Figure S18. Proposed mechanisms for HCOOH.

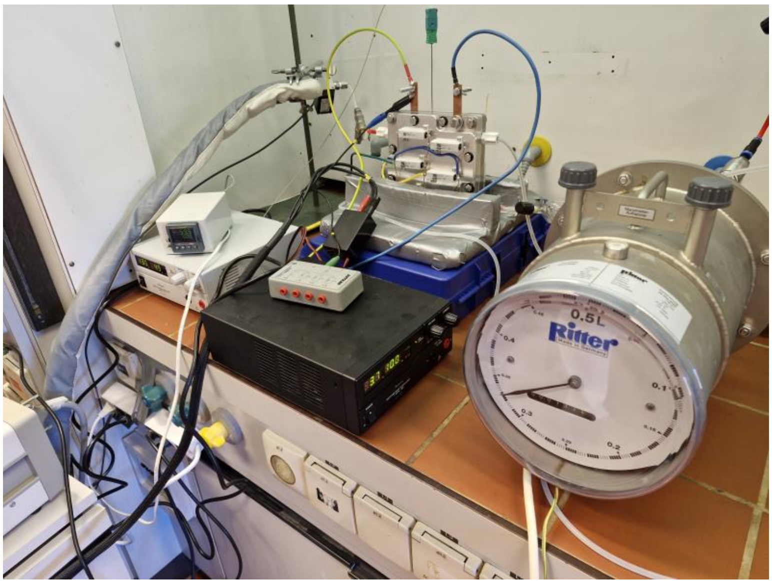

Figure S19. Lab-scale zero-gap electrolyzer equipment.

(1) Pellumbi, K.; Krisch, D.; Rettenmaier, C.; Awada, H.; Sun, H.; Song, L.; Sanden, S. A.; Hoof, L.; Messing, L.; Puring, K. j.; et al. Pushing the Ag-loading of CO2 electrolyzers to the minimum via molecularly tuned environments. *Cell Reports Physical Science* **2023**, *4* (12), 101746. DOI: https://doi.org/10.1016/j.xcrp.2023.101746.
